# Use of dexmedetomidine for prophylactic analgesia and sedation in delayed extubation patients after craniotomy: a study protocol and statistical analysis plan for a randomized controlled trial

**DOI:** 10.1186/1745-6215-14-251

**Published:** 2013-08-13

**Authors:** Li-Hong Zhao, Zhong-Hua Shi, Ning-Ning Yin, Jian-Xin Zhou

**Affiliations:** 1 Department of Critical Care Medicine, Beijing Tiantan Hospital, Capital Medical University, No 6, Tiantan Xili, Dongcheng district, Beijing 100050, China

**Keywords:** Dexmedetomidine, Analgesia, Sedation, Prophylactic, Delayed extubation, Craniotomy

## Abstract

**Background:**

Pain and agitation are common in patients after craniotomy. They can result in tachycardia, hypertension, immunosuppression, increased catecholamine production and increased oxygen consumption. Dexmedetomidine, an alpha-2 agonist, provides adequate sedation without respiratory depression, while facilitating frequent neurological evaluation.

**Methods/design:**

The study is a prospective, randomized, double-blind, controlled, parallel-group design. Consecutive patients are randomly assigned to one of the two treatment study groups, labeled ‘Dex group’ or ‘Saline group.’ Dexmedetomidine group patients receive a continuous infusion of 0.6 μg/kg/h (10 ug/ml). Placebo group patients receive a maintenance infusion of 0.9% sodium chloride for injection at a volume and rate equal to that of dexmedetomidine. The mean percentages of time in optimal sedation, vital signs, various and adverse events, the percentage of patients requiring propofol for rescue to achieve/maintain targeted sedation (Sedation-Agitation Scale, SAS 3 to 4) and total dose of propofol required throughout the study drug infusion are collected. The percentage of patients requiring fentanyl for additional rescue to analgesia and total dose of fentanyl required are recorded. The effects of dexmedetomidine on hemodynamic and recovery responses during extubation are measured. Intensive care unit and hospital length of stay also are collected. Plasma levels of epinephrine, norepinephrine, dopamine, cortisol, neuron-specific enolase and S100-B are measured before infusion (T1), at two hours (T2), four hours (T3) and eight hours (T4) after infusion and at the end of infusion (T5) in 20 patients in each group.

**Discussion:**

The study has been initiated as planned in July 2012. One interim analysis advised continuation of the trial. The study will be completed in July 2013.

**Trial registration:**

ClinicalTrials (NCT): ChiCTR-PRC-12002903.

## Background

Pain and agitation are major concerns in postoperative care of neurosurgical patients. Several studies have shown that moderate-to-severe postoperative pain is frequent following craniotomy [1-7]. In a recent study by Mordhorst *et al*. [2], 55% of patients had moderate or severe postoperative pain in the first 24 hours following craniotomy. This study had a similar result to the pilot study by De Benedittis *et al*. [3] in which 60% of patients experienced postoperative pain. Agitation is common in the ICU. It has been found that there are multiple possible causes of agitation in the ICU setting, including the presence of existing diseases, pain, anxiety, delirium, dressing changes, invasive procedures, nursing procedures, intubation, endotracheal tubes and surgical incisions [8,9]. Pain and agitation can cause tachycardia, hypertension, increased catecholamine production, increased oxygen consumption and immunosuppression [9,10]. Increased catecholamines have been shown to contribute to myocardial ischemia, disturbed sleep and catabolism [11]. Hypertension can cause an increase in brain edema or hemorrhage, which may give rise to brain herniation [12].

On the other hand, despite a greater awareness of pain and agitation after craniotomy, clinicians remain reluctant to administer analgesics and sedatives in patients following craniotomy. The major concerns are the side effects of these drugs, mainly that they reduce the clinician’s ability to monitor the level of consciousness and that they induce respiratory depression [13].

Sedation goals for neurosurgical ICU patients are to improve comfort, overcome anxiety and pain, facilitate nursing care and mechanical ventilation, facilitate frequent neurological assessments and provide sedation without causing deleterious changes in intracranial pressure (ICP) or cerebral perfusion pressure (CPP). An ideal sedative agent in the ICU should be cheap, rapid in onset and offset and without local and systemic adverse effects. There is no sedative agent in use that has all these ideal properties. However, dexmedetomidine, an alpha-2 agonist, provides adequate sedation without respiratory depression, while facilitating frequent neurological evaluation.

Dexmedetomidine, a potent and highly selective alpha-2-adrenoceptor agonist, has been available in the United States since the end of 1999 for use in humans as a short-term (<24 hours) medication for sedation/analgesia in the ICU in initially intubated and mechanically ventilated patients [14].

Dexmedetomidine provides dose-dependent sedation, anxiolysis and analgesia (involving spinal and supraspinal sites) without respiratory depression [15]. Although alpha-2-adrenoceptors are located throughout the body, they are present in larger concentrations in vascular smooth muscle and in key arousal areas of the central nervous system (CNS), such as the locus ceruleus [16]. Activation of alpha-2-adrenoceptors in the CNS decreases centrally mediated sympathetic activity and induces sedation [16,17]. Activation of presynaptic alpha-2-adrenoceptors in cortical blood vessels decreases norepinephrine release, whereas postsynaptic alpha-2-adrenoceptors may directly increase vascular smooth muscle tone [15-17].

Sedation with dexmedetomidine differs from sedation with other commonly used sedatives such as propofol or midazolam. Dexmedetomidine may induce a sedative state similar to physiologic sleep without respiratory depression by acting on alpha-2 receptors in the locus ceruleus [18-20], and patients may be aroused easily with stimulation and are cooperative once aroused.

In addition to its sedative effects, dexmedetomidine has significant analgesic qualities and may significantly reduce concomitant opioid use. Analgesia with dexmedetomidine is mediated primarily through interaction at alpha-2a within the spinal cord, where drug activity attenuates nociceptive signal transduction. The actual mechanism of action appears to involve an interaction with opioid receptors and, although dexmedetomidine alone has been documented to reduce pain, the effect when given jointly with opioids may be additive or synergistic [21]. Dexmedetomidine provides intense analgesia during the postoperative period and reduces the total number of post-surgical patients requiring opioids with a corresponding reduction in opioid-associated side effects [19].

Dexmedetomidine is accompanied by virtually no respiratory depression at clinically relevant doses. Despite profound sedative properties, dexmedetomidine is associated with only limited respiratory effects, even when dosed to plasma levels up to 15 times those normally achieved during therapy, leading to a wide safety margin [18]. Hypercapnic arousal is preserved, and the apnea threshold is actually decreased. Because dexmedetomidine has no depressant effects on ventilation, its analgesic effect may offer a significant advantage for patients at risk of respiratory decompensation [22].

Hemodynamic effects of dexmedetomidine may result from both peripheral and central mechanisms. Dexmedetomidine is the pharmacologically active dextroisomer of medetomidine [23]. Stimulation of alpha-2 adrenoceptors by dexemedetomidine in pontine locus ceruleus (LC) results in decreased firing of LC neurons secondary to their hyperpolarization. It also has a sympatholytic effect through decreases in the concentration of norepinephrine. This, in turn, decreases the blood pressure (BP) and the heart rate (HR) [24-27]. The initial response to rapid dexmedetomidine infusion may be a transient hypertension. However, the net effect of alpha-2 adrenoceptor action is a significant reduction in circulating catecholamines and a modest decrease in BP and heart rate HR. Although hypotension has been described in patients receiving dexmedetomidine, this physiological effect seems to correlate with the use of a loading dose and/or pre-existing hypovolemia [15].

A meta-analysis including 24 trials showed that dexmedetomidine did not appear to increase risk of bradycardia. The risk of bradycardia was, however, significantly higher in studies that used both a loading dose and high maintenance doses (>0.7 ug/kg/h) than in studies that did not use both. Risks of hypotension requiring interventions, delirium, self-extubation, myocardial infarction, hyperglycemia, atrial fibrillation and mortality were not significantly different between dexmedetomidine and traditional sedative and analgesic agents [28].

Dexmedetomidine could be a potentially useful anesthetic adjuvant for neurosurgical patients [15]. Two studies have examined its use in patients undergoing intracranial surgery [29,30]. Both studies showed that the addition of dexmedetomidine improves perioperative hemodynamic control. In these studies, the authors controlled arterial blood pressure with a prescribed incremental dosing of anesthetics. No patient in either study required antihypertensive medications. Dexmedetomidine does not have a significant effect on intracranial pressure [15].

Dexmedetomidine also minimizes opioid induced muscle rigidity, decreases post-operative shivering and has hemodynamic stabilizing effects [31]. The medication is a unique sedative in that it does not cause respiratory depression and the sedated patients are easily aroused to perform a neurological examination or to co-operate with procedures (for example, physiotherapy, radiology imaging) without showing irritation [32,33].

Dexmedetomedine has been reported to provide neuroprotective effects against ischemia in the CNS [34-38]. The S100B protein and the neuron-specific enolase (NSE) are the most widely investigated of molecular markers in patients with severe head injury, stroke or subarachnoid hemorrhage (SAH). The concentration of these markers has been shown to increase in the cerebrospinal fluid (CSF) as well as in the serum [39-43]. In addition, in patients with SAH the course of the S100B concentration has been shown to correlate with neurologic deficits and outcome [44,45].

The aim of the present work is to evaluate the safety and efficacy of dexmedetomidine for prophylactic analgesia and sedation in delayed extubation patients after craniotomy. The primary hypothesis is that prophylactic use of dexmedetomidine will increase the percentage of time in optimal sedation.

## Methods/design

The present study is a prospective, single-center, randomized, double-blind, placebo-controlled, two-arm trial in patients with delayed extubation after craniotomy.

### Study setting and population

The study setting is the neurosurgical ICU, Beijing Tiantan Hospital, Capital Medical University, Beijing, China.

All patients after intracranial surgery with delayed extubation admitted to our ICU are screened daily for study eligibility. Patients are deemed eligible for this study if they are ≥18-years-old, present with motor responses to external stimuli in the Glasgow Coma Scale (GCS-M) equal or higher than five in two hours (time zero would be the time of ICU admission).

Exclusion criteria are:

• Liver insufficiency

• Renal insufficiency

• HR less than 50 bpm

• Systolic BP less than 90 mm Hg

• Acute myocardial infarction

• Second or third degree atrioventricular block

• Need of continuous infusions of vasopressor before the start of study drug infusion

• Consciousness disorders or epilepsy before intracranial surgery

• Pre-existent dysphagia and cough reflex difference

• Emergency operation

• Readmitted to ICU after randomization to the study

• Enrolled in another trial

• Pregnant or lactating women

### Ethical aspects and informed consent

When a patient is identified as eligible for the study, immediate contact will be made with the 24-hour on-call study coordinator, who will confirm eligibility. Delayed extubation patients after craniotomy are often unable to provide consent until they improve. The attending physician will introduce the family to the study coordinator. The physician will make sure the family knows the credentials of the study coordinator, and say that this person is going to discuss a research program that is being conducted, and that this person is qualified to do so. The study coordinator will take the family to a place where they can talk confidentially. Every relevant aspect of the project will be described. The study coordinator will stop frequently, ask if there are any questions, and request that the family repeat back in their own words what is being discussed, to make sure they understand.

The study coordinator will explain that there is a possibility that the patient may experience pain, agitation, and delirium,and, if so, the patient could become worse. The coordinator will say that there is a new alpha-2 agonist that provides adequate sedation without respiratory depression, while facilitating frequent neurological evaluation. He will explain that in a small percentage of patients, dexmedetomidine could cause bradycardia, hypotension and respiratory depression. The potential advantages of using or not using dexmedetomidine will be described. The study coordinator will be especially careful to assure the family that they are free to decline consent without consequences and that they can withdraw consent at any time without impact on treatment. Family members will be provided with contact information for the study coordinator, local co-investigator and the local Ethical Committee. Written consent will be obtained in the presence of a witness.

A register is kept of all patients evaluated for inclusion and of patients who withdraw from the study. The latter are clinically followed up without their data being analyzed in the study.

The study protocol and consent forms were approved by the Institutional Review Board of Beijing Tiantan Hospital Affiliated to Capital Medical University (approval number KY2012-006-02) and by the Chinese Clinical Trial Registry (ChiCTR-PRC-12002903). The Beijing Tiantan Hospitals’ Institutional Review Board gave positive advice for the addition of their ICU as the study site.

### Data collected at study entry

At baseline, data on demographic characteristics and the history of past illnesses of the patients are obtained. The surgical site, operation time, intraoperative medication, the input liquid quantity, and the amount of bleeding and urine are recorded. The Acute Physiology and Chronic Health Evaluation II score (APACHE II) and Glasgow Coma Scale (GCS) are calculated.

### Randomization, double-blind and allocation concealment

The study has a prospective, randomized, double-blind, controlled, parallel-group design. Consecutive patients are randomly assigned to one of the two treatment study groups, labeled ‘Dex group’ or ‘Saline group.’ Randomization is based on a computer generated random digits table and follows a concealed process using sealed and numbered envelopes that allocate the patient to either of the two arms of the study. Patients may be randomized into this study only once unless they were discharged from the hospital and were re-admitted beyond 180 days of the first enrollment. The study does not allow cross-overs and, if any occur, they will be reported as protocol violations.

Experimental drug and placebo with the same character are prepared by a pharmacist. Patients and all study personnel except the investigative pharmacist are blind to treatment assignment. The details of the series are unknown to any of the investigators and are contained in a set of opaque and sealed envelopes, each bearing on the outside only the number.

### Trial interventions

All patients are randomized 1:1 to receive dexmedetomidine or placebo infusion. Dexmedetomidine group patients receive a continuous infusion of 0.6 μg/kg/h (10 ug/ml). Placebo group patients receive a maintenance infusion of 0.9% sodium chloride for injection at a volume and rate equal to that of dexmedetomidine. The patient’s level of sedation will be assessed using the Sedation-Agitation Scale (SAS) per hour after starting the study drug infusion.

Any patient >5 on the SAS will be given rescue propofol in 0.5 mg/kg bolus or infusion doses 0.5 to 2 mg/kg/h until the attaining SAS 3 or 4. Fentanyl will be given in 0.05 mg increments to treat pain as needed.

### Duration of the intervention

The allocated intervention is continued until any of the following criteria is met:

• A maximum of 24 hours on the study infusion protocol

• 30 minutes after extubation

• ICU discharge

The intervention must be emergency stopped when the following occurs:

• HR <50 bpm after atropine in 0.25 mg bolus

• Systolic BP <90 mmHg after hydroxyethyl starch infusion 250 ml/0.5 h

• Peripheral oxygen saturation ( SpO_2_) <90%

• SAS <3

### Study endpoints

#### *The primary endpoints*

Our current primary endpoint is the mean percentages of time in optimal sedation (SAS of 3 or 4).

#### *Secondary endpoints*

The percentage of patients requiring propofol for rescue to achieve/maintain targeted sedation (SAS 3 or 4) and total dose of propofol required throughout the study drug infusion are collected. The percentage of patients requiring fentanyl for additional rescue to analgesia and total dose of fentanyl required are recorded.

Secondary endpoints also include vital signs and various and adverse events. Vital signs are recorded every hour throughout the procedure (BP, HR, respiratory rate (RR), and SpO_2_) .Adverse events include hypotension, hypertension, bradycardia, tachycardia, airway obstruction, apnea, epilepsy, cerebral hemorrhage or infarction and consciousness disorders (GCS-M <5).

The effects of dexmedetomidine on hemodynamic and recovery responses during extubation are measured. BP, HR, RR, and SpO_2_ are recorded one minute before extubation, during extubation, and 1, 3, 5, 10 and 30 minutes after extubation. ICU and hospital length of stay (LOS) are also collected.

Plasma levels of epinephrine, norepinephrine, dopamine, cortisol, NSE and S100-B are measured before infusion (T1), two hours (T2), four hours (T3) and eight hours (T4) after infusion and end of infusion (T5) in 20 patients each group.

### Current sample size justification

Primarily, we expect the frequency of the patient’s agitation to decrease and a reduction of pain after dexmedetomidine infusion in delayed extubation patients after craniotomy. In our previous studies, the baseline frequency of agitation and pain in delayed extubation patients after craniotomy was 30% and the mean percentage of time in optimal sedation was 88%. It is expected that the mean percentages of time in optimal sedation would increase to 95% after dexmedetomidine infusion. Using the Power and Sample Size Calculation program, we will need to study 72 experimental subjects and 72 control subjects to be able to reject the null hypothesis that the population means of the experimental and control groups are equal with a probability (power) of 0.8. The Type I error probability with testing this null hypothesis is 0.05.

### Statistical analysis

All analyses will be according to the intention-to-treat principle, that is, all randomized patients will be analyzed in the groups to which they were originally allocated and will be blinded to treatment assignment. Baseline characteristics will be summarized by carrying out univariate analyses. Categorical variables will be presented as numbers and percents, percentages, and analyzed by the χ^2^-test. Continuous variables will be checked for normal distribution and presented as mean and standard deviation or median and interquartile range as appropriate. Comparison of continuous variables will be performed using Student’s t-test for normally distributed variables and the Mann–Whitney U test for non-normally distributed variables. Plasma levels of epinephrine, norepinephrine, dopamine, cortisol, NSE and S100-β, and BP, HR, RR and SpO_2_ will be analyzed by repeated measure analysis of variance (ANOVA). All tests of significance will be at the 5% significance level and two-sided. Analyses will be conducted using SPSS 17.0.

## Discussion

A significant difference in the safety and/or efficacy endpoints will provide important evidence for optimizing intracranial surgery patient sedation. Also, a neutral result will provide important insight, as this would mean that more studies are needed to evaluate the safety and efficacy of dexmedetomidine for prophylactic analgesia and sedation in patients after craniotomy.

## Trial status

The study has been initiated as planned in July 2012. One interim analysis advised continuation of the trial. The study will be completed in July 2013.

## Abbreviations

APACHE II: Acute Physiology and Chronic Health Evaluation II score; BP: blood pressure; CNS: central nervous system; CPP: cerebral perfusion pressure; CSF: cerebrospinal fluid; GCS: Glasgow Coma Scale; GCS-M: motor responses to external stimuli in GCS; HR: heart rate; ICP: intracranial pressure; LC: locus ceruleus; NSE: neuron-specific enolase; RR: respiratory rate; SAH: subarachnoid hemorrhage; SAS: Sedation-Agitation Scale; SpO2: peripheral oxygen saturation.

## Competing interests

The authors declare that they have no competing interests.

## Authors’ contributions

LHZ and JXZ participated in the design of the study and drafted the manuscript. ZHS and NNY participated in the design of the study. All authors edited the manuscript and read and approved the final manuscript.

## References

[B1] Nemergut EC Durieux ME Missaghi NB Himmelseher S Pain management after craniotomy Best Pract Res Clin Anaesthesiol 2007 21 557 573 10.1016/j.bpa.2007.06.005 18286837

[B2] Mordhorst C Latz B Kerz T Wisser G Schmidt A Schneider A Jahn-Eimermacher A Werner C Engelhard K Prospective assessment of postoperative pain after craniotomy J Neurosurg Anesthesiol 2010 22 202 206 10.1097/ANA.0b013e3181df0600 20479664

[B3] De Benedittis G Lorenzetti A Migliore M Spagnoli D Tiberio F Villani RM Postoperative pain in neurosurgery: a pilot study in brain surgery Neurosurgery 1996 38 466 469 8837797 10.1097/00006123-199603000-00008

[B4] Quiney N Cooper R Stoneham M Walters F Pain after craniotomy. A time for reappraisal? Br J Neurosurg 1996 10 295 299 10.1080/02688699650040179 8799542

[B5] Klimek M Ubben JF Ammann J Borner U Klein J Verbrugge SJ Pain in neurosurgically treated patients: a prospective observational study J Neurosurg 2006 104 350 359 10.3171/jns.2006.104.3.350 16572646

[B6] Thibault M Girard F Moumdjian R Chouinard P Boudreault D Ruel M Craniotomy site influences postoperative pain following neurosurgical procedures: a retrospective study Can J Anaesth 2007 54 544 548 10.1007/BF03022318 17602040

[B7] Gottschalk A Berkow LC Stevens RD Mirski M Thompson RE White ED Weingart JD Long DM Yaster M Prospective evaluation of pain and analgesic use following major elective intracranial surgery J Neurosurg 2007 106 210 216 10.3171/jns.2007.106.2.210 17410701

[B8] Fraser GL Prato BS Riker RR Berthiaume D Wilkins ML Frequency, severity, and treatment of agitation in young versus elderly patients in the ICU Pharmacotherapy 2000 20 75 82 10.1592/phco.20.1.75.34663 10641977

[B9] Barr J Fraser GL Puntillo K Ely EW Gélinas C Dasta JF Davidson JE Devlin JW Kress JP Joffe AM Coursin DB Herr DL Tung A Robinson BR Fontaine DK Ramsay MA Riker RR Sessler CN Pun B Skrobik Y Jaeschke R American College of Critical Care Medicine Clinical practice guidelines for the management of pain, agitation, and delirium in adult patients in the intensive care unit Crit Care Med 2013 41 263 306 23269131 10.1097/CCM.0b013e3182783b72

[B10] Gehlbach BK Kress JP Sedation in the intensive care unit Curr Opin Crit Care 2002 8 290 298 10.1097/00075198-200208000-00004 12386488

[B11] Epstein J Breslow MJ The stress response of critical illness Crit Care Clin 1999 15 17 33 10.1016/S0749-0704(05)70037-3 9929784

[B12] Basali A Mascha EJ Kalfas I Schubert A Relation between perioperative hypertension and intracranial hemorrhage after craniotomy Anesthesiology 2000 93 48 54 10.1097/00000542-200007000-00012 10861145

[B13] Beretta L De Vitis A Grandi E Sedation in neurocritical patients: is it useful? Minerva Anestesiol 2011 77 828 834 21730931

[B14] Jacobi J Fraser GL Coursin DB Riker RR Fontaine D Wittbrodt ET Chalfin DB Masica MF Bjerke HS Coplin WM Crippen DW Fuchs BD Kelleher RM Marik PE Nasraway SA Jr Murray MJ Peruzzi WT Lumb PD Task Force of the American College of Critical Care Medicine (ACCM) of the Society of Critical Care Medicine (SCCM), American Society of Health-System Pharmacists (ASHP), American College of Chest Physicians Clinical practice guidelines for the sustained use of sedatives and analgesics in the critically ill adult Crit Care Med 2002 30 119 141 10.1097/00003246-200201000-00020 11902253

[B15] Bekker A Sturaitis MK Dexmedetomidine for neurological surgery Neurosurgery 2005 57 Suppl 1 1 10 15987564 10.1227/01.neu.0000163476.42034.a1

[B16] Prielipp RC Wall MH Tobin JR Groban L Cannon MA Fahey FH Gage HD Stump DA James RL Bennett J Butterworth J Dexmedetomidine-induced sedation in volunteers decreases regional and global cerebral blood flow Anesth Analg 2002 95 1052 1059 12351293 10.1097/00000539-200210000-00048

[B17] Gerlach AT Dasta JF Dexmedetomidine: an updated review Ann Pharmacother 2007 41 245 254 10.1345/aph.1H314 17299013

[B18] Venn RM Hell J Grounds RM Respiratory effects of dexmedetomidine in the surgical patient requiring intensive care Crit Care 2000 4 302 308 10.1186/cc712 11056756 PMC29047

[B19] Venn RM Bradshaw CJ Spencer R Realey D Caudwell E Naughton C Vedio A Singer M Feneck R Treacher D Willatts SM Grounds RM Preliminary UK experience of dexmedetomidine, a novel agent for postoperative sedation in the intensive care unit Anaesthesia 1999 54 1136 1142 10.1046/j.1365-2044.1999.01114.x 10594409

[B20] Huupponen E Maksimow A Lapinlampi P Särkelä M Saastamoinen A Snapir A Scheinin H Scheinin M Meriläinen P Himanen SL Jääskeläinen S Electroencephalogram spindle activity during dexmedetomidine sedation and physiological sleep Acta Anaesthesiol Scand 2008 52 289 294 10.1111/j.1399-6576.2007.01537.x 18005372

[B21] Fairbanks CA Stone LS Kitto KF Alpha-2c adrenergic receptors mediate spinal analgesia and adrenergic-opioid synergy J Pharmacol Exp Ther 2002 300 282 290 10.1124/jpet.300.1.282 11752127

[B22] Talke P Chen R Thomas B Aggarwall A Gottlieb A Thorborg P Heard S Cheung A Son SL Kallio A The haemodynamic and adrenergic effects of perioperative dexmedetomidine infusion after vascular surgery Anesth Analg 2000 90 834 839 10.1213/00000539-200004000-00011 10735784

[B23] Nelson LE Lu J Guo T Saper CB Franks NP Maze M The alpha 2-adrenoceptor agonist dexmedetomidine converges on an endogenous sleep-promoting pathway to exert its sedative effects Anesthesiology 2003 98 428 436 10.1097/00000542-200302000-00024 12552203

[B24] Ebert TJ Hall JE Barney JA Uhrich TD Colinco MD The effects of increasing plasma concentrations of dexmedetomidine in humans Anesthesiology 2000 93 382 394 10.1097/00000542-200008000-00016 10910487

[B25] Turan G Ozgultekin A Turan C Dincer E Yuksel G Advantageous effects of dexmedetomidine on haemodynamic and recovery responses during extubation for intracranial surgery Eur J Anaesthesiol 2008 25 816 820 10.1017/S0265021508004201 18400140

[B26] Chrysostomou C Schmitt CG Dexmedetomidine: sedation, analgesia and beyond Expert Opin Drug Metab Toxicol 2008 4 619 627 10.1517/17425255.4.5.619 18484919

[B27] Bekker AY Basile J Gold M Riles T Adelman M Cuff G Mathew JP Goldberg JD Dexmedetomidine for awake carotid endarterectomy: efficacy, hemodynamic profile, and side effects J Neurosurg Anesthesiol 2004 16 126 135 10.1097/00008506-200404000-00004 15021281

[B28] Tan JA Ho KM Use of dexmedetomidine as a sedative and analgesic agent in critically ill adult patients: a meta-analysis Intensive Care Med 2010 36 926 939 10.1007/s00134-010-1877-6 20376429

[B29] Soliman RN Hassan AR Rashwan AM Omar AM Prospective, randomized study to assess the role of dexmedetomidine in patients with supratentorial tumors undergoing craniotomy under general anaesthesia Middle East J Anesthesiol 2011 21 325 334 22428485

[B30] Tanskanen P Kytta J Randell T Aantaa R Dexmedetomidine as an anaesthetic adjuvant in patients undergoing intracranial tumor surgery: a double-blind, randomized and placebo-controlled study Br J Anaesth 2006 97 658 665 10.1093/bja/ael220 16914460

[B31] Weinbroum AA Ben-Abraham R Dextromethorphan and dexmedetomidine: new agents for the control of perioperative pain Eur J Surg 2001 167 563 569 10.1080/110241501753171146 11716440

[B32] Tobias JD Berkenbosch JW Sedation during mechanical ventilation in infants and children: dexmedetomidine versus midazolam South Med J 2004 97 451 455 10.1097/00007611-200405000-00007 15180019

[B33] Venn RM Grounds RM Comparison between dexmedetomidine and propofol for sedation in the intensive care unit: patient and clinician perceptions Br J Anaesth 2001 87 684 690 10.1093/bja/87.5.684 11878517

[B34] Kuhmonen J Polorny J Miettinen R Haapalinna A Jolkkonen J Riekkinen P Sr Sivenius J Neuroprotective effects of dexmedetomidine in the gerbil hippocampus after transient global ischemia Anesthesiology 1997 87 371 377 10.1097/00000542-199708000-00025 9286902

[B35] Maier C Steinberg GK Sun GH Zhi GT Maze M Neuroprotection by the alpha 2-adrenoreceptor agonist dexmedetomidine in a focal model of cerebral ischemia Anesthesiology 1993 79 306 312 10.1097/00000542-199308000-00016 8102042

[B36] Hoffman WE Kochs E Werner C Thomas C Albrecht RF Dexmedetomidine improves neurologic outcome from incomplete ischemia in the rat Anesthesiology 1991 75 328 332 10.1097/00000542-199108000-00022 1677549

[B37] Cosar M Eser O Fidan H Sahin O Buyukbas S Ela Y Yagmurca M Ozen OA The neuroprotective effect of dexmedetomidine in the hippocampus of rabbits after subarachnoid hemorrhage Surg Neurol 2009 71 54 59 10.1016/j.surneu.2007.08.020 18207556

[B38] Eser O Fidan H Sahin O Cosar M Yaman M Mollaoglu H Songur A Buyukbas S The influence of dexmedetomidine on ischemic rat hippocampus Brain Res 2008 1218 250 256 18514174 10.1016/j.brainres.2008.04.045

[B39] Pleines UE Morganti-Kossmann MC Rancan M Joller H Trentz O Kossmann T S-100 beta reflects the extent of injury and outcome, whereas neuronal specific enolase is a better indicator of neuroinflammation in patients with severe traumatic brain injury J Neurotrauma 2001 18 491 498 10.1089/089771501300227297 11393252

[B40] Raabe A Kopetsch O Woszczyk A Lang J Gerlach R Zimmermann M Seifert V Serum S-100B protein as a molecular marker in severe traumatic brain injury Restor Neurol Neurosci 2003 21 159 169 14530578

[B41] Rothoerl RD Woertgen C Brawanski A S-100 serum levels and outcome after severe head injury Acta Neurochir Suppl 2000 76 97 100 11450101 10.1007/978-3-7091-6346-7_20

[B42] Woertgen C Rothoerl RD Brawanski A Early S-100B serum level correlates to quality of life in patients after severe head injury Brain Inj 2002 16 807 816 10.1080/02699050210128933 12217206

[B43] Woertgen C Rothoerl RD Metz C Brawanski A Comparison of clinical, radiologic, and serum marker as prognostic factors after severe head injury J Quant Spectrosc Radiat Transf 1999 47 1126 1130 10.1097/00005373-199912000-00026 10608545

[B44] Sanchez-Peña P Pereira AR Sourour NA Biondi A Lejean L Colonne C Boch AL Al Hawari M Abdennour L Puybasset L S100B as an additional prognostic marker in subarachnoid aneurysmal hemorrhage Crit Care Med 2008 36 2267 2273 10.1097/CCM.0b013e3181809750 18596638

[B45] Weiss N Sanchez-Peña P Roche S Beaudeux JL Colonne C Coriat P Puybasset L Prognosis value of plasma S100B protein levels after subarachnoid aneurysmal hemorrhage Anesthesiology 2006 104 658 666 10.1097/00000542-200604000-00008 16571959

